# Characterization and Antibacterial Activity of a Low-Molecular-Weight Bacteriocin-like Inhibitory Substance S-2 Produced by *Leuconostoc falkenbergense* SBL-85-2 Against *Aeromonas hydrophila*

**DOI:** 10.3390/vetsci13090933

**Published:** 2026-09-09

**Authors:** Binglun Sui, Boran Zhang, Yuqi Wang, Bowen Lou, Cheng Jiang, Wanli Sha, Wenlong Dong, Baishuang Yin

**Affiliations:** 1 College of Animal Science and Technology, Jilin Agricultural Science and Technology College, Jilin 132101, China; 19806196391@163.com (B.S.); 13569857101@163.com (B.Z.); momodebql666@163.com (Y.W.); 13364593430@163.com (B.L.); jiangcheng@jlnku.edu.cn (C.J.); shawanli@jlnku.edu.cn (W.S.); 2 Jilin Provincial Key Laboratory of Preventive Veterinary Medicine, Jilin Agricultural Science and Technology College, Jilin 132101, China; 3 Jilin Province Technology Innovation Center of Pig Ecological Breeding and Disease Prevention and Control, Jilin Agricultural Science and Technology College, Jilin 132101, China; 4 Jilin Province Cross Regional Cooperation Technology Innovation Center of Porcine Main Disease Prevention and Control, Jilin 132101, China

**Keywords:** *Leuconostoc falkenbergense*, *Aeromonas hydrophila*, bacteriocin-like inhibitory substance, antibacterial activity

## Abstract

*Aeromonas hydrophila* is a globally distributed aquatic pathogen. Antibiotic overuse in traditional management practices has intensified problems such as antimicrobial resistance and environmental pollution, creating an urgent need for safe alternatives. This study characterized a BLIS produced by *Leuconostoc falkenbergense* SBL-85-2, which shows potential as an antibiotic alternative in aquaculture. *L. falkenbergense* SBL-85-2 exhibited no hemolytic activity, lacked typical high-risk virulence factors, and only low-identity vancomycin-related intrinsic genes (*vanT* and *vanY*) were identified by the CARD database, which may preliminarily indicate its potential biosafety for aquatic applications. The BLIS exhibited marked activity against *A. hydrophila*, with an inhibition zone of 39.14 ± 0.65 mm, as well as other critical pathogens including *Aeromonas rivipollensis*, *Aeromonas salmonicida*, *Escherichia coli*, *Salmonella Typhimurium*, *Staphylococcus aureus*, and *Vagococcus fluvialis*. Furthermore, the BLIS exhibited remarkable thermal stability (retained 87.18 ± 0.14% activity after treatment at 100 °C for 10 min) and UV stability (92.67 ± 0.75% residual activity after 3 h exposure) coupled with sensitivity to proteases, confirming its proteinaceous nature. Molecular weight determination indicated that the BLIS is a low-molecular-weight peptide (<2.7 kDa). Mechanistically, the BLIS disrupted bacterial cell membrane integrity, ultimately resulting in the leakage of cellular contents and a time-dependent reduction in intracellular ATP levels (reduced by 69.16 ± 4.10% at 2 h), exerting its antimicrobial effect. Collectively, our results indicate that this BLIS combines potent antibacterial efficacy with a favorable biosafety profile, suggesting its significant potential for the biological control of *A. hydrophila* in aquaculture.

## Simple Summary

This study aimed to characterize BLIS S-2, a bacteriocin-like inhibitory substance produced by lactic acid bacteria, and evaluate its potential against the aquatic pathogen *Aeromonas hydrophila*. Experiments confirmed that this substance is heat-stable, non-toxic, and exerts antibacterial activity through disruption of the outer membrane. Collectively, these findings provide a novel strategy for controlling *A. hydrophila* in aquaculture and offer a promising direction for reducing antibiotic dependence and developing safe, effective alternatives.

## 1. Introduction

*Aeromonas hydrophila* is a facultatively anaerobic, Gram-negative opportunistic aquatic pathogen that is widely distributed in rivers, lakes, estuaries, and various wastewater systems, and persists in drinking water and groundwater [1,2,3,4,5,6,7,8]. It has an extremely broad host range, encompassing fish [9], amphibians [10], reptiles [11], birds [12], and mammals [13], and can cause motile aeromonas sepsis (MAS) [14], red spot disease [15], and other *Aeromonas*-associated diseases characterized by high morbidity and mortality rates, posing a substantial threat to global aquaculture production. Historically, *A. hydrophila* outbreaks have caused massive fish mortality worldwide, resulting in significant economic losses [16]; furthermore, its detection in marine species and human hosts in recent years has raised additional public health concerns regarding food safety and zoonotic diseases [17,18].

Traditionally, the prevention and control of *A. hydrophila* infections have relied heavily on antibiotics; however, this has exacerbated antimicrobial resistance (AMR) [19], which not only undermines treatment efficacy but also poses a challenge to global public health security. Therefore, developing safe, effective, and sustainable prevention and control strategies has become a top priority. Bacteriocin-like inhibitory substances (BLIS) are a class of ribosomally synthesized antimicrobial peptides produced by various bacteria. Among these, BLIS produced by lactic acid bacteria (LAB) are considered highly promising alternatives to antibiotics due to their high specificity against multidrug-resistant (MDR) pathogens and their generally recognized as safe (GRAS) status [20,21].

*L. falkenbergense* is a food-grade lactic acid bacterium commonly used in fermented foods and food preservation [22]. This species belongs to the genus *Leuconostoc* spp. and was formally named in 2021, with strains isolated from various fermented foods such as fermented green beans and yogurt [23]. It is considered safe and its genome has been shown to contain putative bacteriocin biosynthetic gene clusters [24]. Although LAB have been extensively studied for their applications in the food industry [25], the potential of *L. falkenbergense* as a novel antimicrobial peptide-producing strain remains largely unexplored. Among antimicrobial peptides produced by LAB, class II bacteriocins have attracted considerable attention due to their potent inhibitory activity against foodborne pathogens such as *Listeria monocytogenes*. These bacteriocins are characterized by remarkable thermal stability and do not require extensive post-translational modifications for activity. *Leuconostoc* spp. have emerged as one of the most important sources of such bacteriocins, and a variety of class II bacteriocins with potent antibacterial activity have been identified from multiple species within this genus. Their molecular masses typically range from 3.5 to 7 kDa, as exemplified by leucocin A (predicted mass ~3.9 kDa) [26] and leucocin C (~5.3 kDa) [27]. Notably, this study shows that *L. falkenbergense* exhibits significant antibacterial activity against *A. hydrophila*, an important opportunistic pathogen in aquaculture, suggesting that the bacteriocins it produces may possess a broader antimicrobial spectrum. This provides new research perspectives for the application of this strain in multiple fields, including food preservation and aquatic disease control.

## 2. Materials and Methods

### 2.1. Isolation and Identification of Bacteria

Strain SBL-85-2 was isolated from the discarded intestines of fish collected from a wet market in Shandong Province, China. After diluting the sample, it was spread onto MRS agar plates and incubated at 37 °C for 24 h. Multiple single colonies with distinct morphological characteristics were selected and purified through repeated streaking. Gram staining was used to verify its purity and morphological characteristics. One of the Gram-positive bacteria was named SBL-85-2; it was mixed with 20% glycerol and stored at −80 °C for subsequent research. Bacterial genomic DNA was extracted using a bacterial genomic DNA kit (Aidlab Biotechnologies Co., Ltd., Beijing, China). The target DNA was amplified via polymerase chain reaction (PCR) using the universal primers 5′-AGAGTTTGATCCTGGCTCAG-3′ and 5′-GGTTACCTTGTTACGACTT-3′ [28]. The samples were then submitted to General Biosystems (Anhui) Co., Ltd. (Chuzhou, China) for 16S rRNA gene sequencing, and the resulting data were compared with the GenBank database. The 16S rRNA gene sequence was deposited in GenBank under the accession number PZ312073.

### 2.2. Hemolytic Activity Assay

In this experiment, the blood agar plate method was used to evaluate the hemolytic activity of *L. falkenbergense* SBL-85-2, which is one of the key virulence indicators for assessing its potential pathogenicity. The blood agar plates used in the experiment were purchased from Bickman Biotechnology Co., Ltd., Changde, Hunan, China. *A. hydrophila* ATCC 7966, which exhibits β-hemolysis, was used as the positive control, and sterile phosphate-buffered saline (PBS) was used as the negative control. After overnight incubation of the test strain SBL-85-2 in MRS broth at 37 °C, 20 µL of the bacterial culture, the positive control culture, and the negative control PBS were each aspirated using a micropipette and spotted onto the surface of blood agar plates. The inoculated plates were incubated at 37 °C for 12 h.

### 2.3. Preparation of CFS and Crude BLIS S-2 from L. falkenbergense

*L. falkenbergense* was inoculated into MRS broth and allowed to activate for 24 h; it was then transferred at a 2% inoculum volume to 300 mL of MRS broth and incubated with shaking at 37 °C for 48 h. The culture was centrifuged at 3625× *g* for 15 min using a TFL16M centrifuge (Yancheng Kate Laboratory Instruments Co., Ltd., Yancheng, China). The resulting supernatant was filtered through a 0.22 µm polyethersulfone microporous membrane to obtain the cell-free supernatant (CFS). The CFS was concentrated tenfold using a rotary evaporator at 50 °C and subsequently dialyzed against deionized water at 4 °C for 24 h using a dialysis membrane with a 3.5 kDa molecular weight cut-off (MWCO). After dialysis, the retentate inside the dialysis bag and the dialysate outside the bag were concentrated separately, and their antibacterial activity was finally evaluated using the agar diffusion method. The fraction exhibiting inhibitory activity was designated as the crude BLIS S-2 and stored at 4 °C until further use.

### 2.4. Antibacterial Activity Assay

To fully eliminate interference from organic acids, the pH of the crude BLIS S-2 was adjusted to 6.0 using NaOH; catalase was then added to achieve a final concentration of 1 mg/mL. After incubating at 37 °C for 2 h, the sample was subsequently heat-treated at 100 °C for 5 min to inactivate the catalase. *A. hydrophila* ATCC 7966 was streaked onto Brain Heart Infusion (BHI) agar plates and incubated at 37 °C for 24 h. Single colonies were then transferred to BHI broth and incubated at 37 °C for 24 h. After incubation, the culture was centrifuged at 7378× *g* for 5 min, washed three times with 0.9% sterile saline solution, resuspended, and the bacterial concentration was adjusted to 1 × 10^6^ CFU/mL. Antibacterial activity was determined using the agar diffusion method. 50 µL of the activated test strain suspension was evenly spread onto a Mueller-Hinton (MH) agar plate. Using an 8-mm Oxford cup, 200 µL of the crude BLIS S-2 was added to wells prepared in the agar, and the plate was left at room temperature for 1 h to allow diffusion and then incubated at 37 °C for 12 h. A catalase-only control (catalase added to MRS broth at 1 mg/mL, incubated at 37 °C for 2 h, and heat-inactivated at 100 °C for 5 min) was included to confirm that the catalase treatment itself did not produce any inhibitory effect on the indicator strain. The diameter of the inhibition zone was measured using a Vernier caliper.

### 2.5. Antimicrobial Spectrum

Subsequently, the antibacterial activity of the crude BLIS S-2 against both animal- and aquaculture-derived pathogens was evaluated using the standardized agar diffusion method under strictly controlled conditions. The test organisms included three animal-derived pathogens (*E. coli* ATCC 25922, *S. Typhimurium* ATCC 14028, and *S. aureus* ATCC 25923) and four aquaculture-derived pathogens (*A. hydrophila* ATCC 7966, *A. rivipollensis* MZ-17, *A. salmonicida* XN-9, and *V. fluvialis* ZXL-80). All indicator bacteria were cultured at 37 °C for 12 h and then inoculated into BHI broth (*A. hydrophila* ATCC 7966, *A. rivipollensis* MZ-17, *A. salmonicida* XN-9, and *V. fluvialis* ZXL-80) and Luria–Bertani (LB) broth (*E. coli* ATCC 25922, *S. aureus* ATCC 25923, and *S. Typhimurium* ATCC 14028), respectively. The concentration of the indicator bacteria was adjusted to 1 × 10^6^ CFU/mL, and the zones of inhibition were measured using a Vernier caliper.

### 2.6. Growth Curve of L. falkenbergense SBL-85-2

*L. falkenbergense* SBL-85-2 was inoculated into 300 mL of MRS broth at a 2% inoculation rate and incubated with shaking at 37 °C for 24 h. A 200 µL sample was collected every 2 h to monitor changes in pH and optical density (OD_600_) at a wavelength of 600 nm. At the same time, 1 mL of the culture was collected, centrifuged at 7378× *g* for 5 min, and then tested for antibacterial activity using the agar diffusion method.

### 2.7. Growth Inhibition Kinetics

*A. hydrophila* ATCC 7966 was cultured in BHI broth at 37 °C until the logarithmic growth phase, then centrifuged at 7378× *g* for 5 min and washed three times with sterile saline. *L. falkenbergense* SBL-85-2 was cultured in MRS broth at 37 °C until the logarithmic growth phase, then centrifuged at 7378× *g* for 5 min to obtain CFS. Twenty milliliters of the indicator strain culture was mixed with 20 mL of CFS and 20 mL of MRS medium, respectively, to form the experimental and control groups, which were then incubated at 37 °C for 24 h. Samples were collected every 2 h, and the growth of *A. hydrophila* ATCC 7966 was monitored by measuring the absorbance at 600 nm (OD_600_).

### 2.8. Molecular Weight Determination

To further determine the molecular weight of the active substance, Tricine-SDS-PAGE was employed for analysis. This method utilizes the Tricine buffer system to optimize separation efficiency, significantly improving the resolution for low-molecular-weight proteins (1–100 kDa, particularly <20 kDa), thereby enabling more precise molecular weight determination. The experiment employed a non-continuous gel system consisting of a 4% stacking gel, a 10% spacer gel, and an 18% separating gel. Protein molecular weight standards ranging from 2.7 to 40 kDa (Sangon Biotech, Shanghai, China) were used as references. Electrophoresis was performed under constant voltage conditions: following the loading of 15 µL of the crude BLIS S-2 and 10 µL of the marker, the run commenced at 30 V until the marker entered the spacer gel. The voltage was then increased to 80 V until the dye front migrated into the separating gel, after which it was raised to 120 V until electrophoresis was complete. After electrophoresis, the gel was fixed in a fixing solution (50% methanol, 10% acetic acid, 40% distilled water) for 30 min. After rinsing with sterile distilled water, the gel was stained with Coomassie Brilliant Blue R-250. Subsequently, the gel was decolorized multiple times with a decolorizing solution (20% ethanol, 10% glacial acetic acid, 70% distilled water) until the gel background was clear and the protein bands were distinct [29].

To identify bands with antibacterial activity in the gel, in situ antibacterial assays were performed using the overlay method. The electrophoresed gel was rinsed three times with sterile distilled water and laid flat on the bottom of a sterile Petri dish. Subsequently, MH agar containing *A. hydrophila* ATCC 7966 (1 × 10^6^ CFU/mL) was poured evenly over the gel surface to completely cover it. The plate was incubated at 37 °C for 12 h, and zones of inhibition formed at the positions corresponding to the gel bands were observed [30,31].

### 2.9. Stability Assays

#### 2.9.1. pH Stability

The BLIS S-2 samples were pre-incubated and adjusted to pH 4.0, 5.0, 6.0, 7.0, 8.0, and 9.0 using 1 mol/L hydrochloric acid or sodium hydroxide, respectively, and incubated in a 37 °C water bath for 2 h. After incubation, all samples were neutralized and adjusted back to the initial pH of 4.3. Antimicrobial activity was then determined using *A. hydrophila* ATCC 7966 as the indicator strain, with the original. The original, pH-untreated BLIS S-2 sample (pH 4.3) was used as the negative control, which underwent the same incubation conditions (37 °C, 2 h) without pH adjustment.

#### 2.9.2. Thermal Stability

The BLIS S-2 samples were heat-treated for 1 h at different temperatures (–20 °C, 4 °C, 37 °C, 60 °C, 80 °C, and 100 °C) and then cooled to room temperature. Antimicrobial activity was determined using *A. hydrophila* ATCC 7966 as the indicator strain. A non-treated BLIS S-2 sample was used as the negative control, representing the baseline condition without thermal exposure.

#### 2.9.3. Protease Stability

The BLIS S-2 samples were carefully adjusted to the optimal pH for each protease using 1 mol/L hydrochloric acid or sodium hydroxide, respectively. Pronase E, trypsin, papain, pepsin, and proteinase K were then added, and the mixture was incubated in a 37 °C water bath for 2 h. The enzymatic reaction was subsequently terminated by heating at 100 °C for 10 min to completely inactivate the proteases. Antimicrobial activity was determined using *A. hydrophila* ATCC 7966 as the indicator strain. A BLIS S-2 sample lacking any protease was used as the negative control, which underwent the same treatment procedures (37 °C, 2 h incubation and 100 °C, 10 min heat-inactivation) without the addition of protease.

#### 2.9.4. UV Stability

The BLIS S-2 samples were exposed to ultraviolet light for up to 0, 0.5, 1, 1.5, 2, 2.5, and 3 h. Antimicrobial activity was then determined using *A. hydrophila* ATCC 7966 as an indicator organism. A BLIS S-2 sample without UV exposure was used as the negative control.

### 2.10. Whole-Genome Sequencing

*L. falkenbergense* SBL-85-2 was inoculated into MRS broth and cultured at 37 °C for 24 h. The cells were harvested by centrifugation, and the resulting cells were sent to Shanghai Majorbio Bio-pharm Technology Co., Ltd. (Shanghai, China) for whole-genome sequencing. Whole-genome sequencing of this strain was performed using a hybrid sequencing approach on the Illumina NovaSeq X Plus and Oxford Nanopore PromethION R9.4 platforms. Raw sequencing data were quality-assessed and filtered using fastp 0.20.0. Subsequently, Unicycler v0.4.8 was used to perform mixed assembly of short-read and long-read data to obtain the complete genome. The final assembly consisted of three circular contigs (one chromosome of 2,120,229 bp and two plasmids of 41,429 bp and 21,013 bp), with a total genome size of 2.18 Mb, a GC content of 39.01%, and an N50 of 2,120,229 bp. Genome completeness and contamination were assessed using CheckM v1.2.5, yielding 100% completeness and 0% contamination. The whole-genome shotgun project of *L. falkenbergense* SBL-85-2 has been deposited in GenBank under accession number JBYIXB000000000, and the raw sequencing reads have been deposited in the Sequence Read Archive (SRA) under accession number SRR38218944. For genome annotation, Prodigal v2.6.3 was used to predict protein-coding genes, while tRNAscan-SE v2.0 and Barrnap v0.9 were used to identify tRNA and rRNA genes, respectively. For bacteriocin synthesis analysis, BAGEL4 v1.2 was used to predict secondary metabolite synthesis gene clusters. BAGEL4 identified a bacteriocin gene cluster on PlasmidA, spanning approximately 17 kb and containing 32 predicted ORFs, among which orf00028 was annotated as pediocin, suggesting a pediocin-like bacteriocin. For comparative genomics and evolutionary analysis, orthomeric gene clustering was performed using OrthoMCL v14-137, and a phylogenetic tree of core genes was constructed to reveal the evolutionary relationships of this strain among BLIS-producing species. Finally, Circos v0.69.6 was used to generate a circular genome map integrating the localization information of the gene clusters.

To systematically evaluate the biosafety of strain SBL-85-2 at the genomic level, prediction analyses of virulence factors and antimicrobial resistance genes were performed. Virulence-related genes were predicted using DIAMOND v0.8.35 (E-value ≤ 1 × 10^−5^) against the VFDB database (version vfdb_v20240301). For resistance gene prediction, resistance gene screening was performed against the CARD database via the Resistance Gene Identifier (RGI) tool.

### 2.11. Live/Dead Cell Staining

To evaluate the antibacterial activity of the crude BLIS S-2 against *A. hydrophila* ATCC 7966, the live/dead cell staining assay was performed to assess cell membrane damage. SYTO 9 is a green fluorescent dye that can pass through intact cell membranes (including those of both live and dead cells), causing the cells to emit green fluorescence; PI, on the other hand, is a red fluorescent dye that can only pass through damaged cell membranes. It competitively displaces SYTO 9 that has already bound to nucleic acids, thereby converting the green fluorescence to red fluorescence. Therefore, in a co-staining system, cells emitting green fluorescence represent live cells with intact cell membranes, while cells emitting red fluorescence represent dead cells with damaged cell membranes. *A. hydrophila* (1 × 10^6^ CFU/mL) was centrifuged at 7378× *g* for 5 min, washed three times with 0.9% sterile saline solution, and then resuspended. The suspension was incubated with an equal volume of BLIS S-2 at 37 °C for 2 h, using a suspension without BLIS S-2 as a negative control. After incubation, the cells were washed three times with 0.9% saline solution, then stained with SYTO 9 and PI for 15 min under light-protected conditions. Finally, live cells (green fluorescence) and dead cells (red fluorescence) were observed under a fluorescence microscope.

### 2.12. Scanning Electron Microscope (SEM)

To investigate the ultrastructural damage caused by the crude preparation of BLIS S-2 to *A. hydrophila* ATCC 7966, *A. hydrophila* ATCC 7966 (1 × 10^6^ CFU/mL) was centrifuged at 7378× *g* for 5 min, washed three times with PBS, and resuspended. The suspension was incubated with an equal volume of BLIS S-2 at 37 °C for 2 h; a suspension treated with an equal volume of PBS served as the control group. After incubation, the cells were washed three times with PBS, centrifuged to collect the cells, and fixed overnight at 4 °C in 2.5% glutaraldehyde. The fixed samples were dehydrated at 4 °C using a series of ethanol gradients (30%, 50%, 70%, 80%, 90%, and 100%) and air-dried. After sputter gold coating, the samples were examined by SEM to observe morphological damage to the cells.

### 2.13. Determination of Intracellular ATP Levels

To determine the effect of the crude BLIS S-2 on *A. hydrophila* ATCC 7966, this experiment measured the dynamic changes in leaking intracellular ATP levels strictly according to the instructions for the Solarbio ATP content assay kit (Beijing, China). *A. hydrophila* ATCC 7966 was cultured to the logarithmic growth phase (at a concentration of 1 × 10^6^ CFU/mL), centrifuged at 7378× *g* for 5 min, washed three times with PBS, and then resuspended. The bacterial suspension was incubated with an equal volume of BLIS S-2 at 37 °C for 0.5 h, 1 h, and 2 h, respectively, with PBS serving as the control group. After incubation, the cells were centrifuged to collect the pellet, washed three times with PBS, and 1 mL of extraction buffer was added. The mixture was then sonicated on an ice bath (parameters: power 200 W, 2 s of sonication followed by 1 s of pause, for a total duration of 1 min). The disrupted solution was centrifuged at 10,000× *g* for 3 min at 4 °C, and the ATP content was finally determined according to the manufacturer’s instructions for the Solarbio (China) ATP Assay Kit.

### 2.14. Statistical Analysis

All experiments were repeated independently three times, and the data are presented as the mean ± standard deviation of the three replicate measurements. Statistical analyses were performed using IBM SPSS Statistics 26.0 software. Prior to the analyses, the Shapiro–Wilk test for normality and Levene’s test for homogeneity of variances were conducted on the data. Under the premise that both assumptions were satisfied, we proceeded to perform one-way analysis of variance (ANOVA), followed by Duncan’s test for post-hoc analysis. A *p*-value of <0.05 was considered statistically significant.

## 3. Results

### 3.1. Phylogenetic Tree

**Figure 1:**
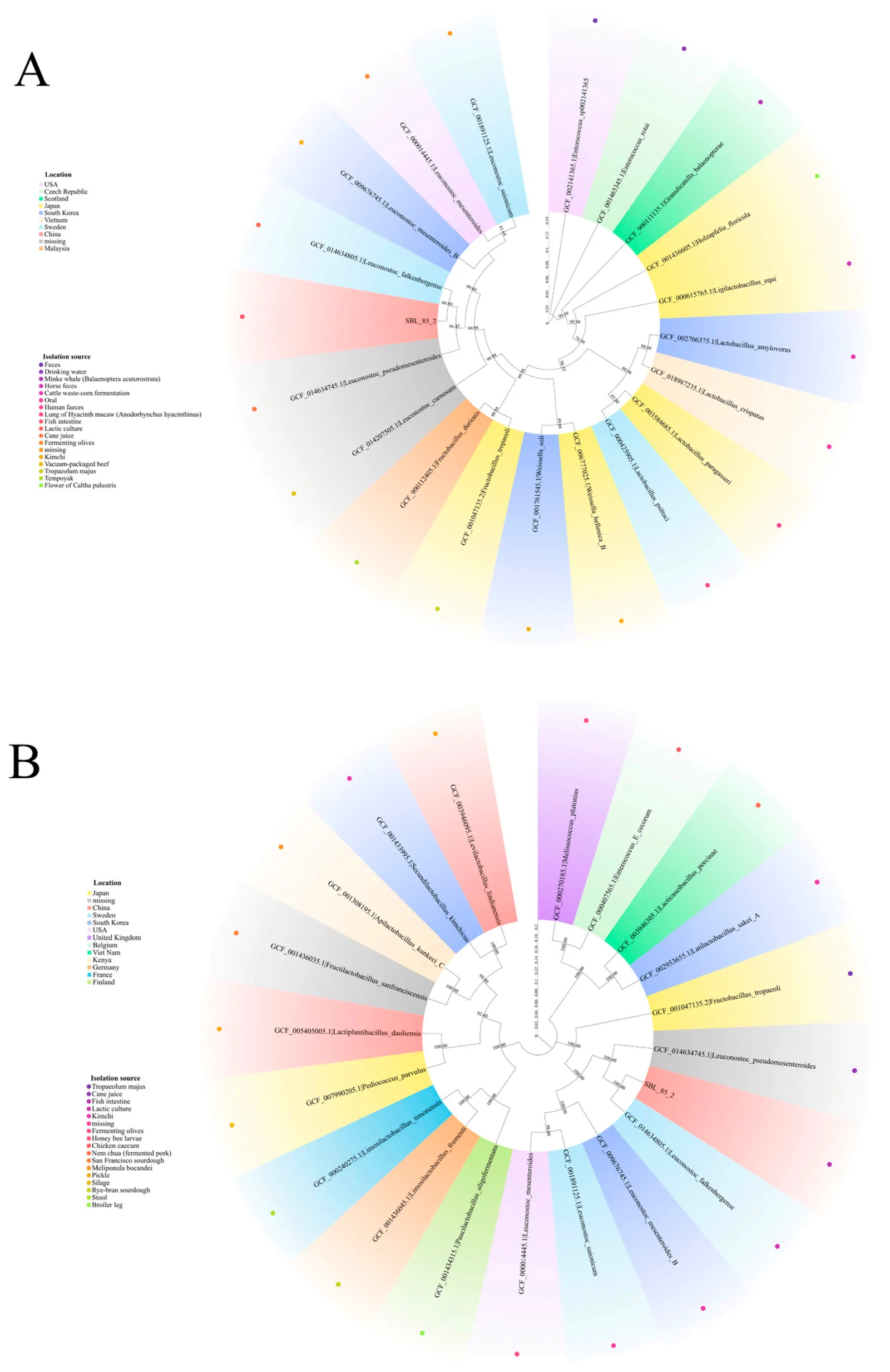
Phylogenetic tree of sample SBL-85-2. Numbers at branch nodes indicate bootstrap values (percentage) calculated from 1000 replicates. (**A**) Phylogenetic tree of SBL-85-2 based on 16S rRNA gene sequences. (**B**) Phylogenetic tree of SBL-85-2 based on 31 housekeeping genes.

A BLAST alignment of 16S rRNA gene sequences revealed that strain SBL-85-2 showed 100% sequence homology with *L. falkenbergense*. To preliminarily determine its phylogenetic relationship, a phylogenetic tree was constructed using 20 strains most closely related to it at the species and genus levels, based on their gene sequences (Figure 1A). To further confirm its taxonomic status with greater accuracy, a phylogenetic tree was constructed based on 31 housekeeping genes (*dnaG*, *frr*, *infC*, *nusA*, *pgk*, *pyrG*, *rplA*, *rplB*, *rplC*, *rplD*, *rplE*, *rplF*, *rplK*, *rplL*, *rplM*, *rplN*, *rplP*, *rplS*, *rplT*, *rpmA*, *rpoB*, *rpsB*, *rpsC*, *rpsE*, *rpsI*, *rpsJ*, *rpsK*, *rpsM*, *rpsS*, *smpB*, *tsf*), a phylogenetic tree was constructed using the 19 closest-related strains at the species and genus levels (Figure 1B). Based on the results of the aforementioned phylogenetic analysis, SBL-85-2 was identified as *L. falkenbergense*.

### 3.2. Hemolytic Activity

**Figure 2:**
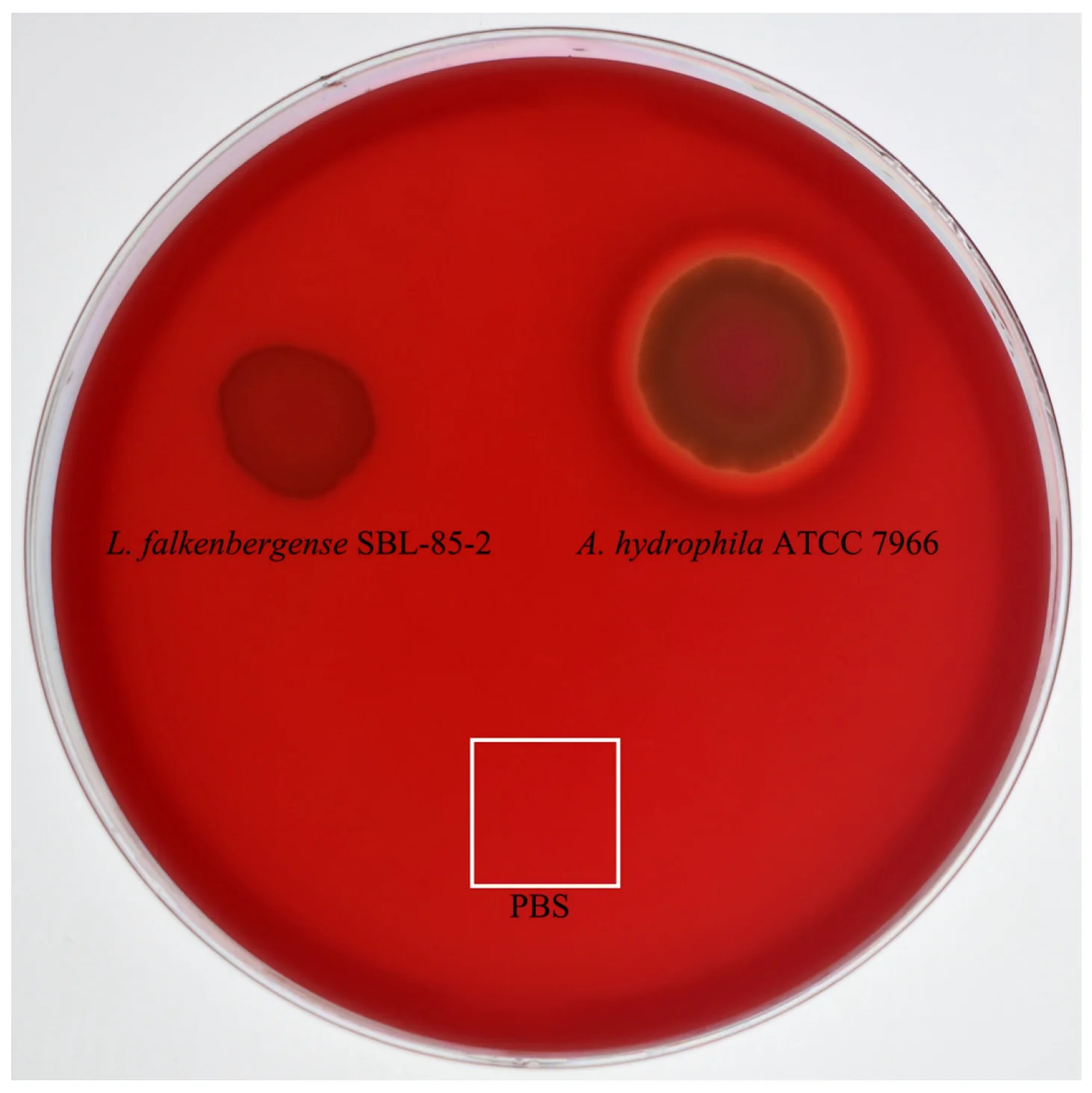
Evaluation of the Hemolytic Safety Profile. The plate shows three inoculation sites: sterile PBS (negative control, highlighted by a white square), *L. falkenbergense* SBL-85-2, and *A. hydrophila* ATCC 7966 (positive control). The PBS droplet contains no bacterial cells and leaves no visible residue after incubation; the inoculation site is therefore indicated by the white square for clarity.

The hemolytic activity of *L. falkenbergense* SBL-85-2 was evaluated using the blood agar plate method. As shown in Figure 2, no hemolytic ring formed around the inoculation site on the agar plate; this phenotype was consistent with that of the sterile PBS negative control and stood in stark contrast to the positive control, *A. hydrophila* ATCC 7966, which exhibited typical β-hemolysis. These results confirm that strain SBL-85-2 lacks hemolytic activity.

### 3.3. Antimicrobial Activity and Antibacterial Spectrum

After eliminating interference from hydrogen peroxide and organic acids, the CFS still retained significant antimicrobial activity, indicating that *L. falkenbergense* SBL-85-2 has the potential to produce BLIS. As shown in Table 1, BLIS-S-2 demonstrated broad-spectrum antimicrobial activity against both Gram-negative and Gram-positive bacteria, with the strongest inhibition against the aquatic pathogen *A. hydrophila* ATCC 7966 (inhibition zone of 39.14 ± 0.65 mm), while inhibition zones against the remaining tested strains ranged from 27.92 ± 0.31 to 37.76 ± 0.47 mm.

### 3.4. Growth Kinetics of L. falkenbergense SBL-85-2 and Growth Inhibition of A. hydrophila ATCC 7966 by CFS

**Figure 3:**
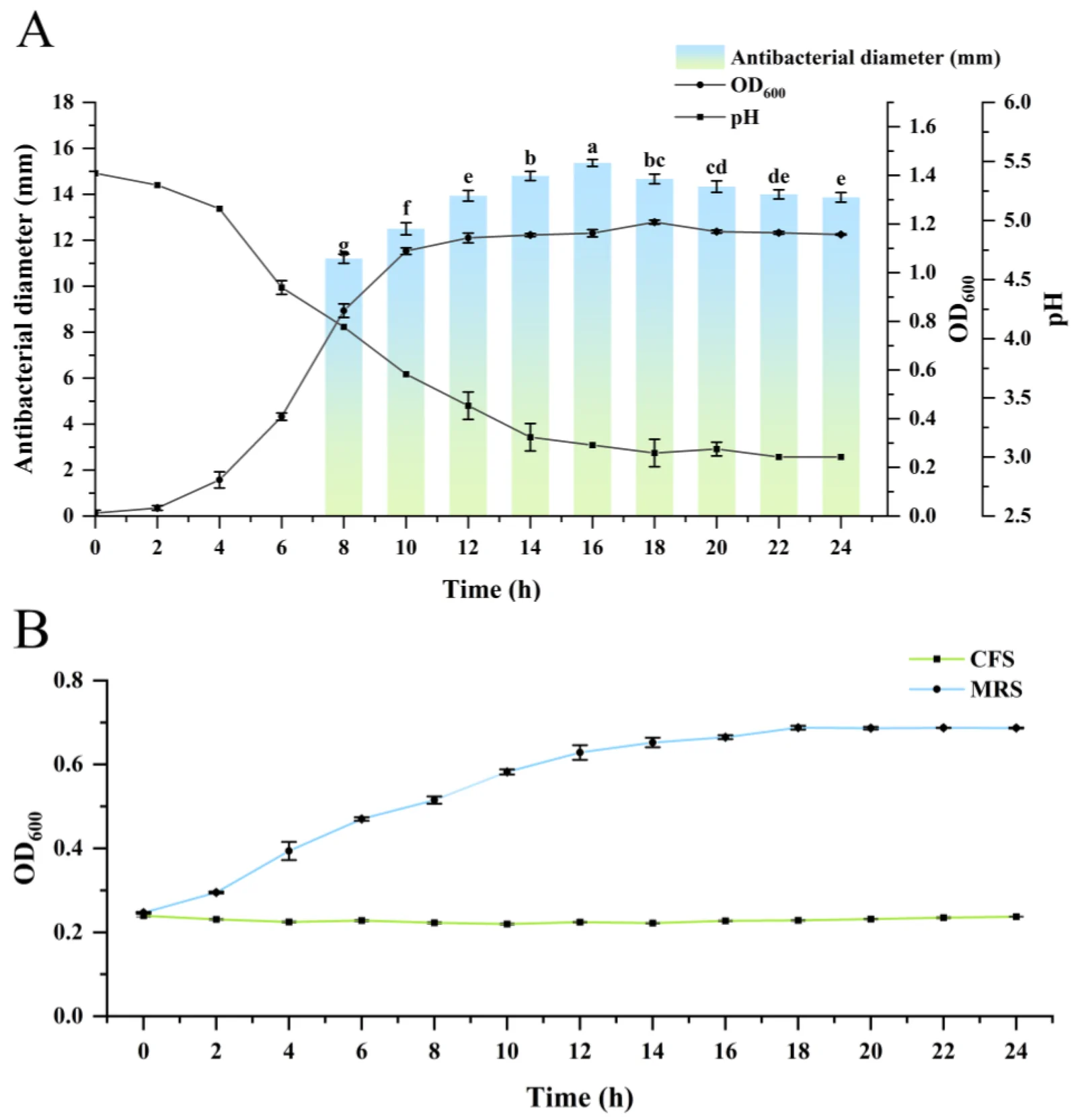
Growth kinetics and antibacterial activity. (**A**) Relationship between incubation time and changes in OD_600_, antibacterial activity, and culture pH. (**B**) Dynamic inhibitory effect of CFS on *A. hydrophila* ATCC 7966. Data are presented as mean ± SD (*n* = 3). Error bars represent standard deviations from three independent replicates. Different lowercase letters above the bars indicate statistically significant differences (*p* < 0.05).

As shown in Figure 3A, the growth of *L. falkenbergense* SBL-85-2 was monitored for 24 h. This strain entered the vigorous logarithmic growth phase after 4 h of incubation and reached the stationary phase after 10 h. Its CFS exhibited antibacterial activity after 8 h of incubation and peaked at 16 h. The pH of the culture continued to decrease starting at 2 h and eventually stabilized at 3.0. As shown in Figure 3B, growth monitoring of the experimental and control groups over a 24 h period revealed that, compared to the MRS broth control group, the growth of *A. hydrophila* ATCC 7966 in the experimental group was significantly inhibited.

### 3.5. Effect of pH, Temperature, Protease and UV on the Antimicrobial Activity

**Figure 4:**
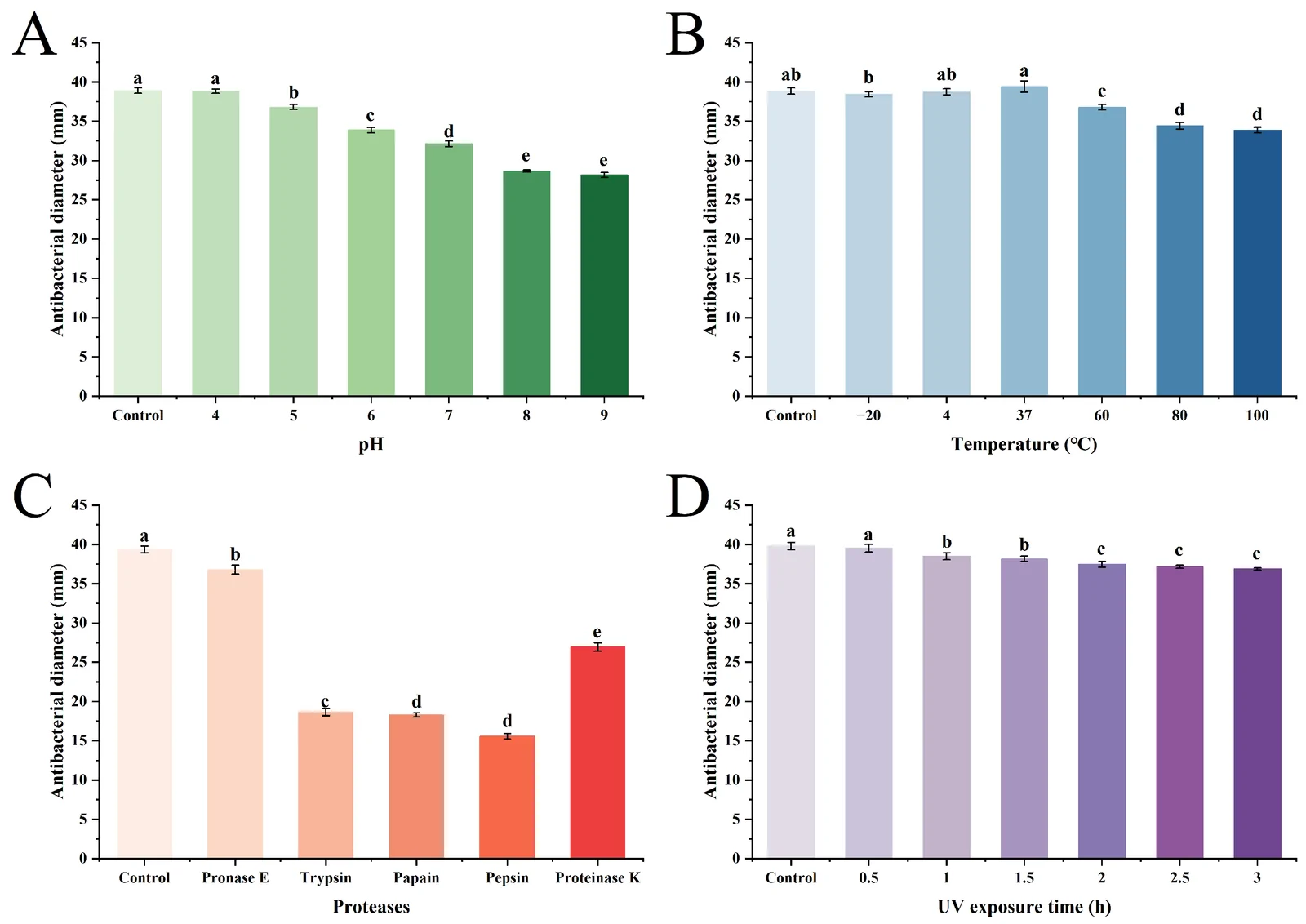
The antimicrobial activity of BLIS S-2 is affected by pH, temperature, proteases, and UV exposure. Antimicrobial activity in all panels is expressed as antibacterial diameter (mm). (**A**) Effect of pH on the antimicrobial activity of BLIS S-2. (**B**) Effect of temperature on the antimicrobial activity of BLIS S-2. (**C**) Effect of proteases on the antimicrobial activity of BLIS S-2. (**D**) Effect of UV exposure on the antimicrobial activity of BLIS S-2. Data are presented as mean ± SD (*n* = 3). Error bars represent standard deviations from three independent replicates. Different lowercase letters above the bars indicate statistically significant differences (*p* < 0.05).

As shown in Figure 4A, the antimicrobial activity of BLIS S-2 decreased significantly as the pH increased. As shown in Figure 4B, BLIS S-2 exhibited excellent thermal stability, retaining 87.18 ± 0.14% of its original activity after treatment at 100 °C for 10 min (*n* = 3). Although statistically significant differences were observed among certain temperature treatments at −20 °C, 4 °C, 37 °C, 60 °C, 80 °C, and 100 °C (as indicated by different lowercase letters in Figure 4B), the overall residual activity remained at a high level, supporting its strong thermal tolerance. Protease sensitivity experiments indicated that BLIS S-2 is sensitive to trypsin (47.36 ± 1.09%), papain (46.43 ± 0.41%), pepsin (39.55 ± 0.54%), and proteinase K (68.42 ± 0.76%), but less sensitive to pronase E (93.42 ± 0.65%) (Figure 4C). As shown in Figure 4D, BLIS S-2 exhibited good tolerance to continuous UV irradiation, retaining 92.67 ± 0.75% residual activity after 3 h exposure (*n* = 3). Although a gradual decrease in antibacterial activity was observed with prolonged exposure time (0–3 h), with statistically significant differences among certain time points (Figure 4D), the activity remained at a functionally effective level throughout the treatment period.

### 3.6. Molecular Weight Analysis and Confirmation

**Figure 5:**
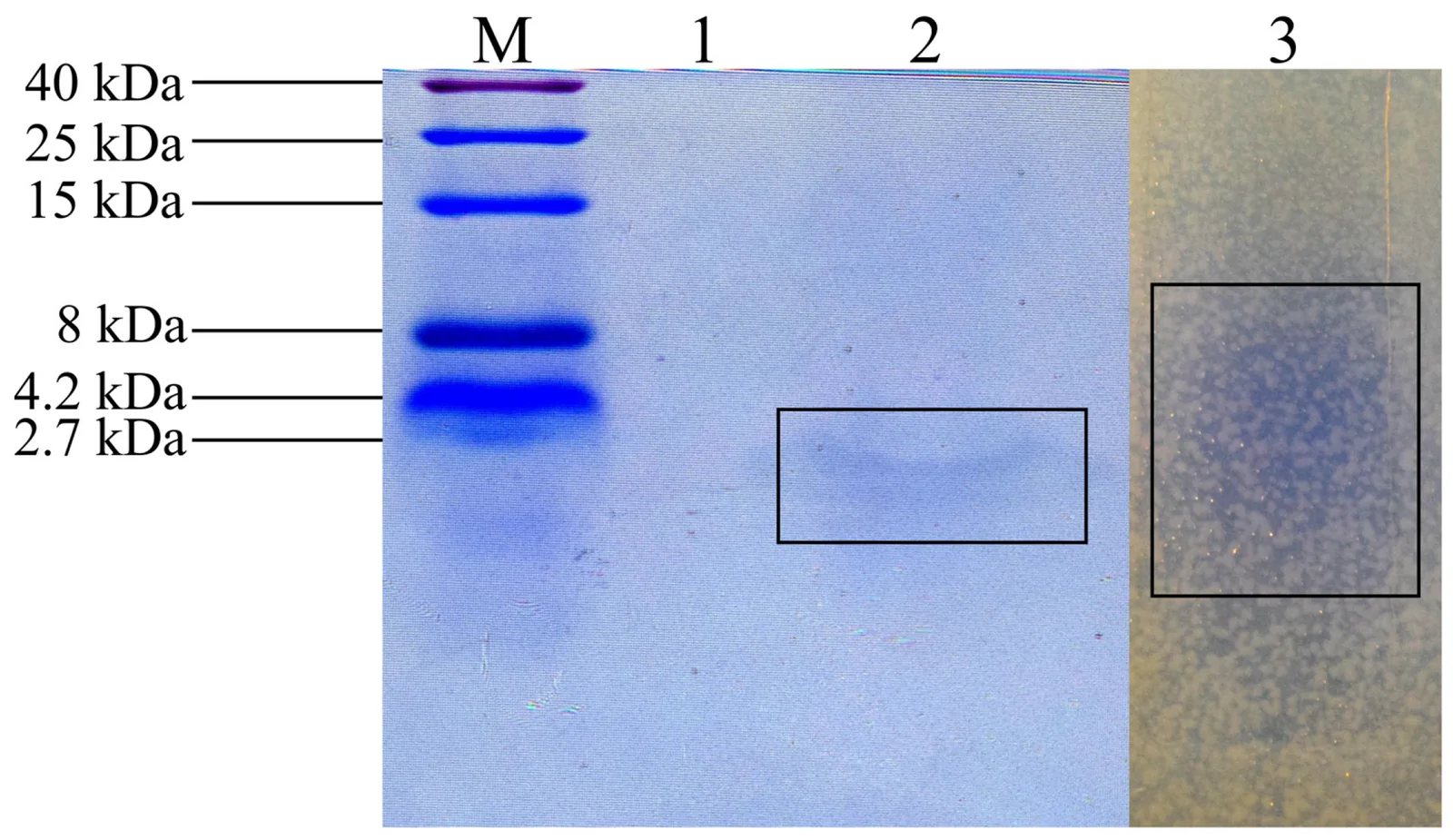
Molecular weight determination of BLIS S-2 by Tricine-SDS-PAGE and identification of the active band by in situ antibacterial overlay assay. Lane M: The first lane of the polyacrylamide gel shows an ultra-low-molecular-weight protein marker. Lane 1: PBS blank control. Lane 2: A distinct single low-molecular-weight protein band corresponding to BLIS S-2 from *L. falkenbergense* SBL-85-2. Lane 3: In situ antibacterial activity assay of BLIS S-2 on agar medium.

After dialysis, the concentrate from the dialysate outside the bag exhibited significant antibacterial activity against the indicator bacteria, preliminarily indicating that BLIS S-2 is a low-molecular-weight substance. Further Tricine-SDS-PAGE analysis revealed a single protein band in the region below 2.7 kDa in the crude BLIS extract (Figure 5). The in situ overlay assay demonstrated that the antibacterial activity co-localized with this <2.7 kDa protein band. Taken together, these results suggest that BLIS S-2 is a low-molecular-weight antimicrobial peptide.

### 3.7. Genomic Characterization and Gene Cluster Analysis of L. falkenbergense SBL-85-2

**Figure 6:**
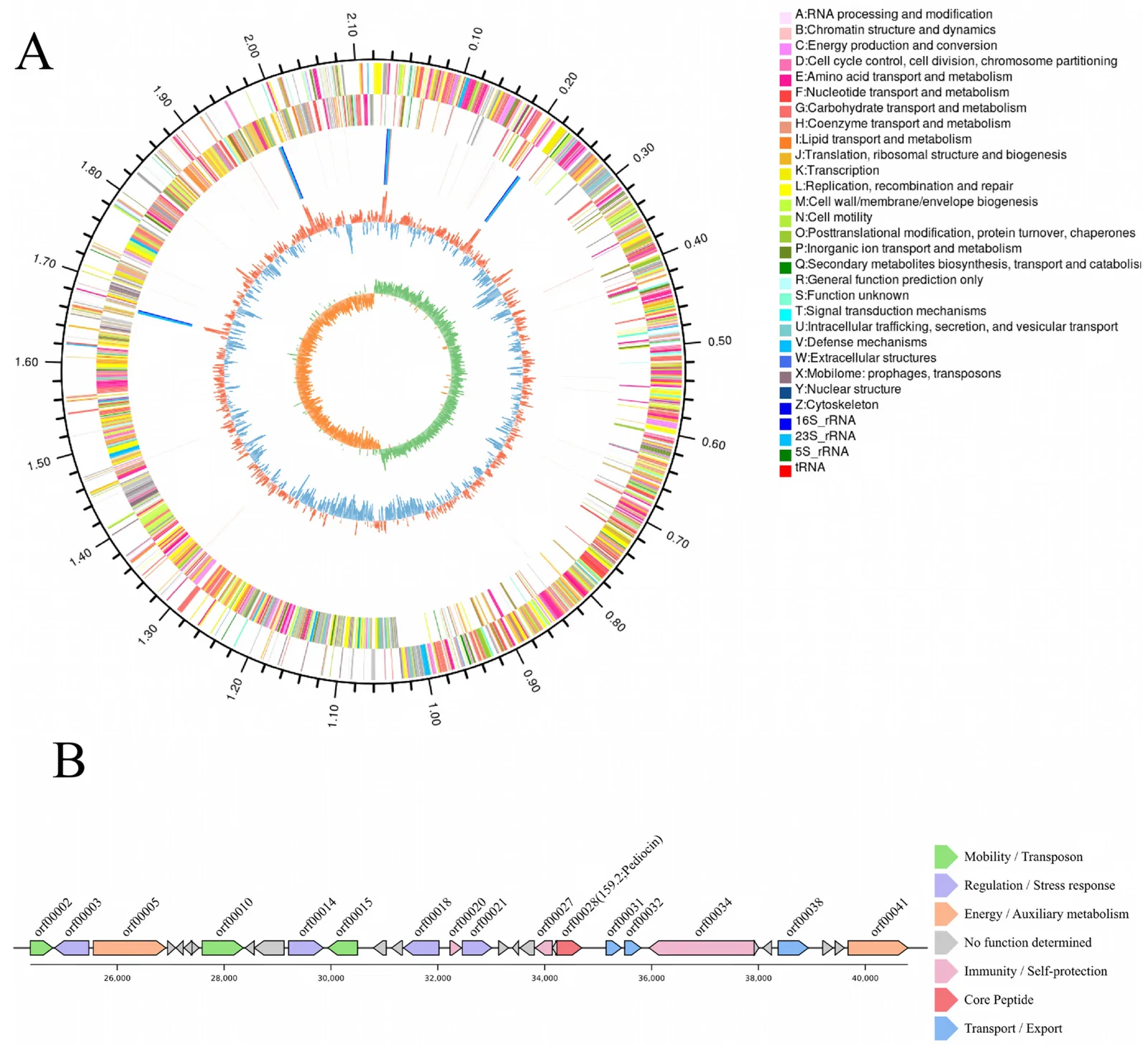
Genomic analysis of *L. falkenbergense* SBL-85-2. (**A**) Circular genome map. The outermost ring indicates the genome size; the second and third rings show CDSs on the positive and negative strands, respectively, with different colors representing the functional classification of CDSs according to COGs; the fourth ring shows rRNA and tRNA; the fifth ring shows GC content; and the innermost ring shows the GC-Skew value. (**B**) Genetic organization of the BLIS gene cluster. Arrows indicate open reading frames (ORFs) and their direction of transcription. ORFs are color-coded according to predicted functional categories: red, core peptide; blue, transport/export; orange, energy/auxiliary metabolism; green, mobility/transposon; purple, regulation/stress response; pink, immunity/self-protection; gray, no function determined.

The genomic circle plot generated using Circos (Figure 6A) comprehensively illustrates its genomic characteristics: the outermost ring indicates the genome size; the second and third rings represent CDSs on the positive and negative strands, respectively, with different colors denoting the functional classifications of CDSs according to COGs; the fourth ring shows rRNAs and tRNAs; the fifth ring displays GC content, with the outward-extending red region indicating that the GC content in that area is higher than the whole-genome average—the higher the peak, the greater the deviation from the average—while the inward-extending blue region indicates that the GC content is lower than the whole-genome average—the higher the peak, the greater the deviation from the average; The innermost ring represents the GC-Skew value, calculated as G-C/G+C. This can aid in distinguishing leading and lagging strands; generally, the leading strand has a GC-Skew > 0, while the lagging strand has a GC-Skew < 0. It can also help identify the replication origin (minimum cumulative offset) and termination site (maximum cumulative offset), which is particularly important for circular genomes.

Whole-genome sequencing and BAGEL4 analysis of strain SBL-85-2 (Figure 6B) suggested the presence of a putative Class II bacteriocin-like biosynthetic gene cluster [32]. The cluster comprises the core structural gene orf00028, encoding a pediocin-like precursor peptide, along with several genes that may be involved in transport (orf00034, orf00038) and metabolism (orf00027, orf00031, orf00032). In addition, multiple transposase-encoding genes for insertion sequences IS905, IS904, and IS3, as well as genes potentially encoding the transcriptional regulator Flp, were also detected within this region.

Virulence-associated gene prediction based on VFDB revealed that the identified genes in *L. falkenbergense* SBL-85-2 were predominantly enriched in functional categories such as Immune modulation and Nutritional/Metabolic factor, and no typical high-risk pathogenic factors were detected, preliminarily confirming the safety of this strain at the genomic level. RGI analysis against the CARD database revealed three strict hits encoding vancomycin resistance-related genes (*vanT* and *vanY*) on the chromosome of SBL-85-2, with % Identity of Matching Region ranging from 32.45% to 36.76%. No resistance genes were detected on the two plasmids.

### 3.8. Bactericidal Activity and Membrane-Damaging Mechanisms of BLIS S-2 on A. hydrophila

**Figure 7:**
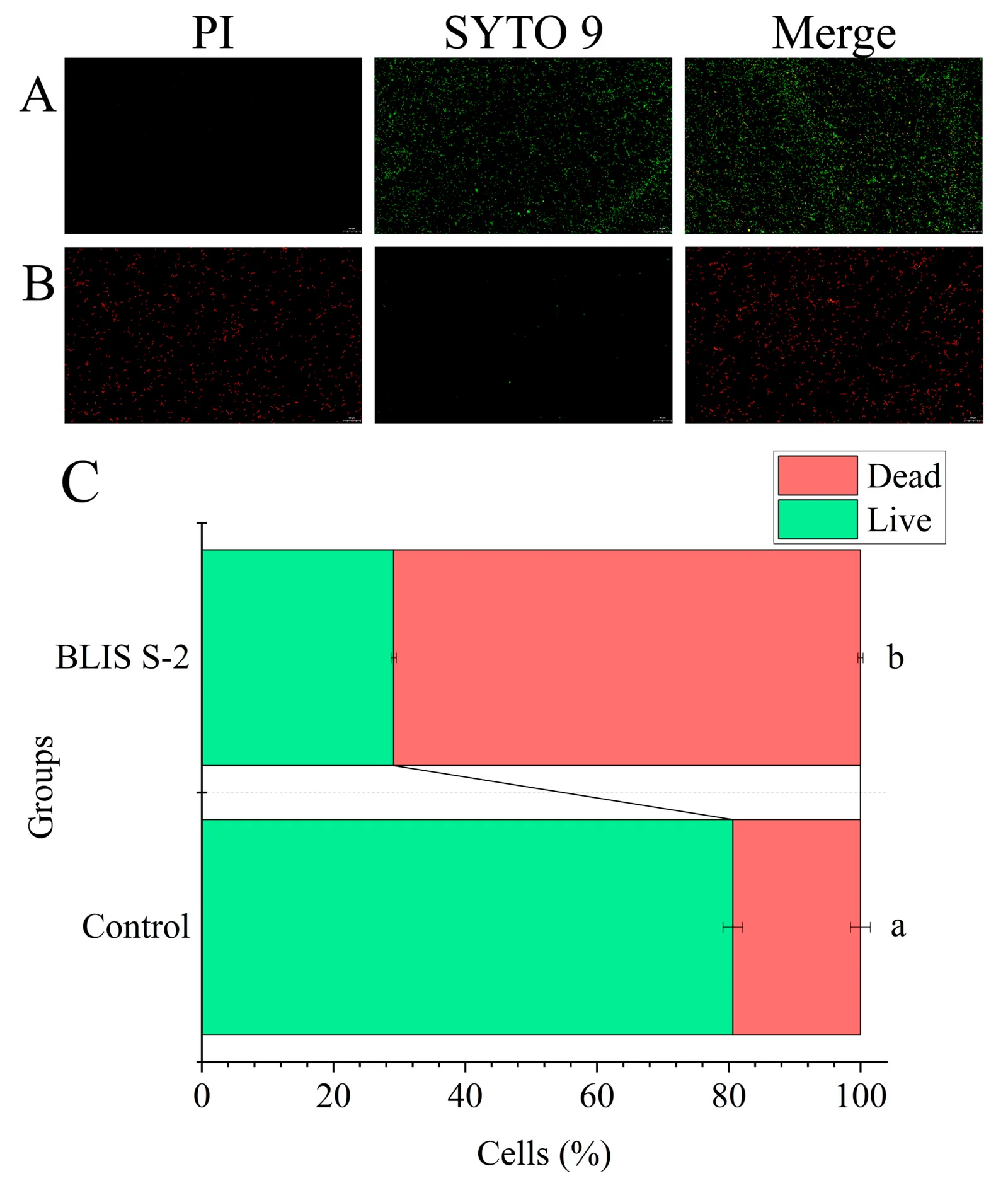
Fluorescence microscopy images of SYTO 9 and PI staining. PI labels dead cells (red), SYTO 9 labels live cells (green), and “Merge” shows the merged image. (**A**) Control group untreated with BLIS S-2. (**B**) Experimental group treated with BLIS S-2. Scale bar: 50 µm; magnification: 30×. (**C**) Quantification of cell viability. The stacked bar chart shows the relative proportions of live (green) and dead (red) cells after 2 h of treatment with BLIS S-2 or 0.9% sterile saline solution control. Data are presented as mean ± SD (*n* = 3). Error bars represent standard deviations from three independent replicates.

The live/dead cell staining assay is shown in Figure 7. The control group exhibited primarily green fluorescence with weak red fluorescence (Figure 7A); in contrast, the experimental group treated with BLIS S-2 showed significantly enhanced red fluorescence (Figure 7B). Based on this, a stacked bar chart was constructed from the quantitative analysis of fluorescence imaging results (Figure 7C). The data presented in this figure clearly demonstrate a significant increase in the percentage of dead cells following BLIS S-2 treatment relative to the control, providing statistical validation of its membrane-damaging effect.

### 3.9. SEM Observation of BLIS S-2-Induced Morphological Damage

**Figure 8:**
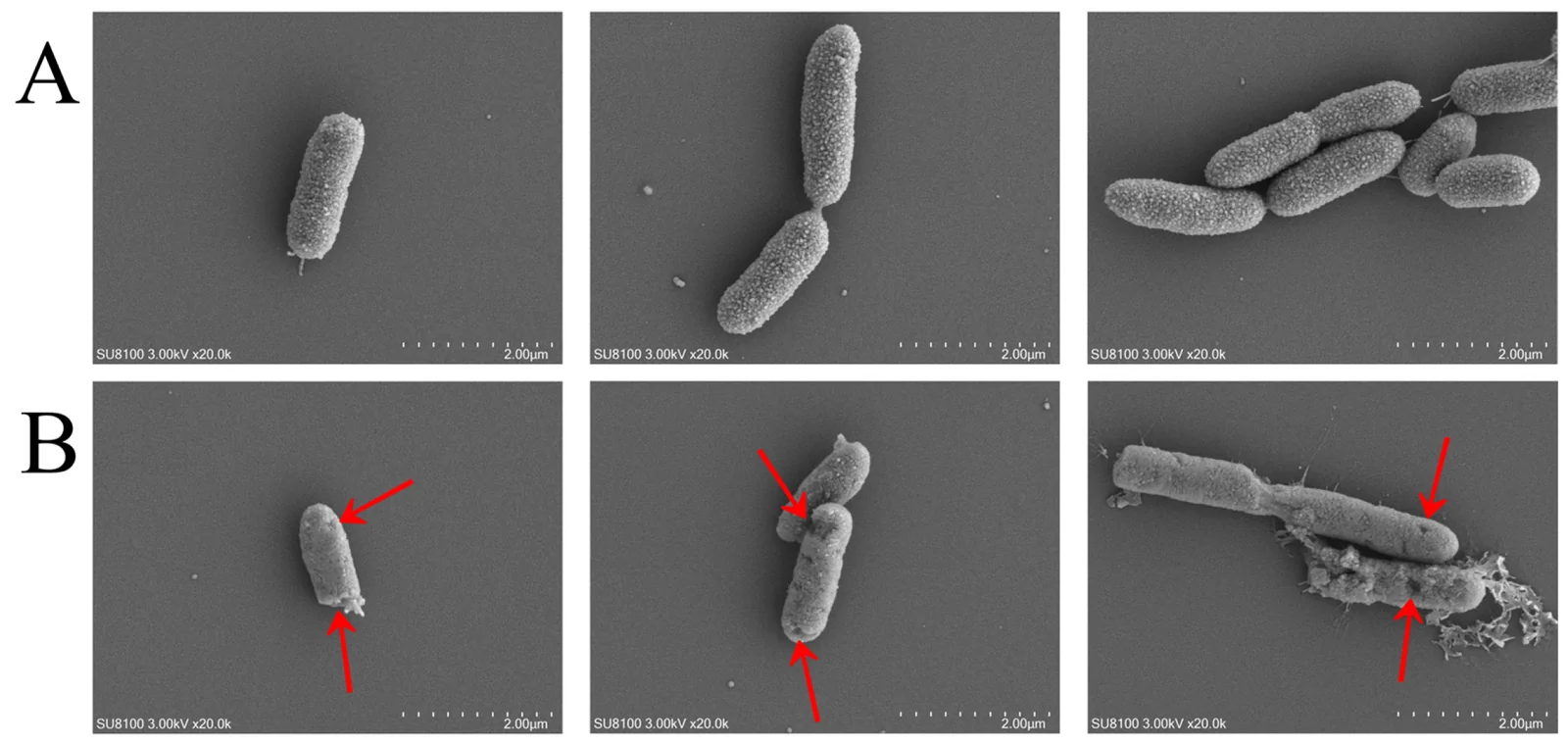
Scanning electron microscopy observations of the effects of BLIS S-2 on the ultrastructure of *A. hydrophila* ATCC 7966 cells. (**A**) Control group with intact cell morphology after PBS treatment. (**B**) Experimental group with damaged cell membrane morphology after BLIS S-2 treatment. Red arrows indicate representative sites of membrane damage. Scale bar: 2.00 µm; magnification: 20.0k×.

Scanning electron microscopy analysis confirmed the antibacterial activity of BLIS S-2 against *A. hydrophila* ATCC 7966 at the morphological level. SEM images clearly show that, compared with the morphologically intact control group (Figure 8A), cells treated with BLIS S-2 exhibited surface roughening, pore formation, and leakage of cellular contents (Figure 8B). These characteristic features of membrane damage directly confirm that the antibacterial activity of BLIS S-2 stems from the disruption of cell membrane integrity.

### 3.10. Intracellular ATP Concentration

**Figure 9:**
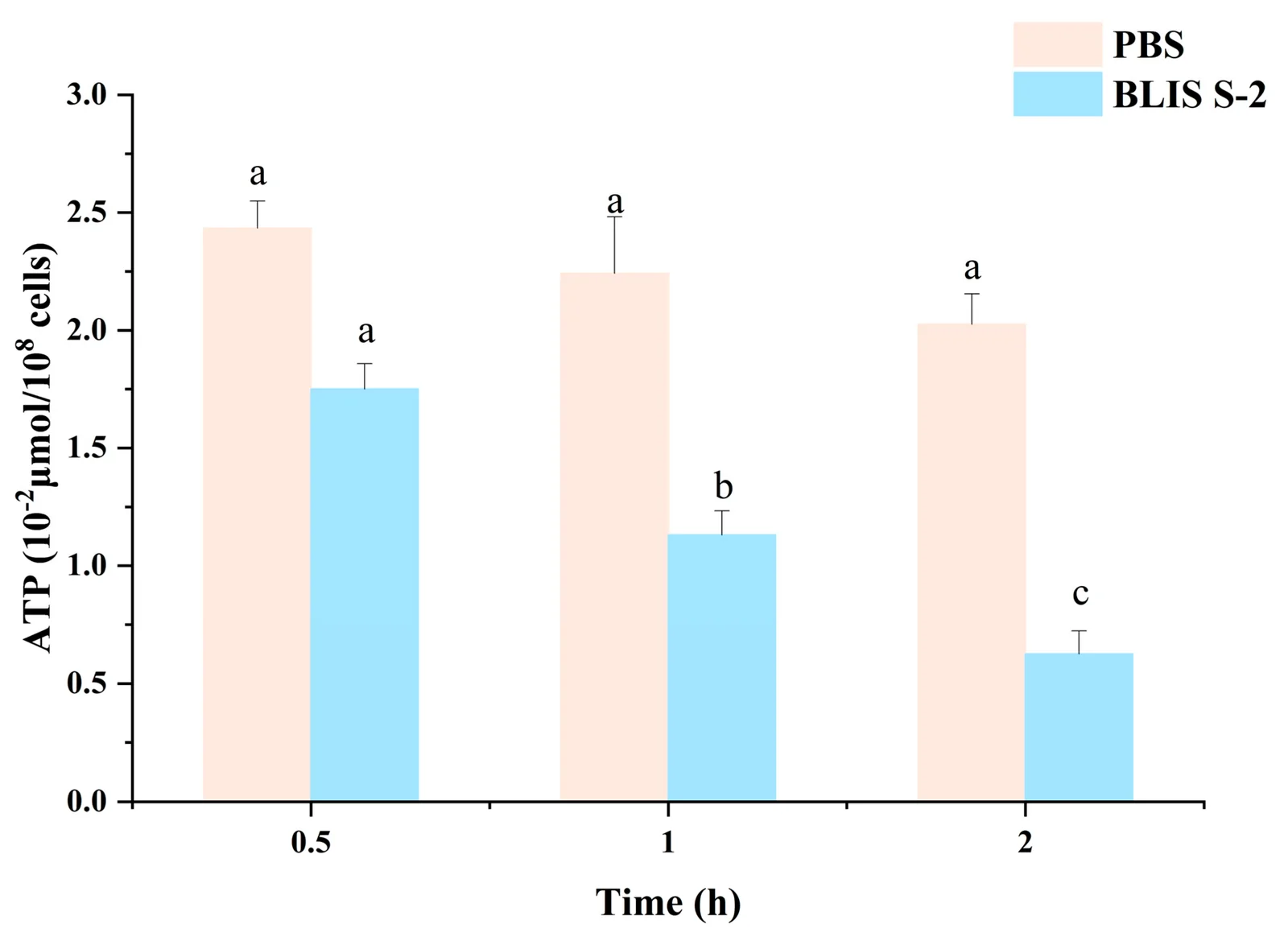
Intracellular ATP levels in *A. hydrophila* ATCC 7966. ATP levels are expressed as 10^−2^ μmol/10^8^ cells. Data are presented as mean ± SD (*n* = 3). Error bars represent standard deviations from three independent replicates. Different lowercase letters above the bars indicate statistically significant differences (*p* < 0.05). The same lowercase letters indicate no significant differences (*p* > 0.05).

The experimental results in Figure 9 show that, as the incubation time increased, the intracellular ATP concentration in the BLIS S-2-treated group exhibited a continuous downward trend compared to the control group, with ATP levels reduced by 69.16 ± 4.10% at 2 h of incubation, indicating a time-dependent inhibitory effect. These results indicate that the BLIS S-2 produced by *L. falkenbergense* SBL-85-2 increases membrane permeability, leading to the leakage of intracellular ATP in *A. hydrophila* ATCC 7966.

## 4. Discussion

In recent years, with the rapid development of the aquaculture industry, diseases caused by pathogens such as *A. hydrophila* have become one of the main factors limiting the industry’s growth, often leading to massive fish mortality and significant economic losses [33]. In the process of disease prevention and control, the overuse of antibiotics not only exacerbates the crisis of antimicrobial resistance (AMR), but also—if therapeutic efficacy is not fully evaluated prior to use—can easily lead to the accumulation of antibiotic residues in the environment and disrupt the balance of aquatic microbial communities [34]. In the face of the growing problem of antibiotic resistance, the development and application of safe and effective new antimicrobial peptides have become particularly urgent. Among these, BLIS produced by LAB, as natural antimicrobial peptides, demonstrate unique application potential.

The results of the hemolytic activity assay showed that *L. falkenbergense* SBL-85-2 did not produce any clear or discolored hemolytic rings around the inoculation site; its phenotype was consistent with that of the negative control, in sharp contrast to the positive control, *A. hydrophila*, which produced typical β-hemolysis. This result is of significant importance: First, it suggests that SBL-85-2 does not secrete hemolysin. Hemolysin is a key virulence factor for many aquatic pathogens (such as certain Vibrio species and *A. hydrophila*); it can lyse host red blood cells, destroy tissues, and help pathogens evade immune clearance. Therefore, the absence of hemolytic activity serves in aquatic applications [35]. Second, these results reveal its excellent biocompatibility at the level of cell membrane interactions. The absence of a hemolytic ring suggests that the metabolites of this strain do not cause nonspecific lysis of eukaryotic cell membranes [36]. We therefore hypothesize that the strain may exhibits low cytotoxicity to fish tissues and may possesses selective toxicity, potentially preferentially targeting bacterial membranes (rich in anionic phospholipids) over eukaryotic membranes (rich in cholesterol and neutral in surface charge). In summary, the negative results for hemolytic activity, combined with its potential BLIS-producing capacity, collectively indicate that *L. falkenbergense* SBL-85-2 is a promising candidate for aquaculture applications, laying an important toxicological foundation for its further evaluation as an eco-friendly biological control agent or probiotic supplement.

To comprehensively evaluate the biosafety of *L. falkenbergense* SBL-85-2 at the genomic level, virulence factors and antimicrobial resistance genes were systematically predicted. Virulence-associated gene prediction based on the VFDB database revealed that the identified genes were predominantly enriched in functional categories associated with immune modulation and nutritional/metabolic factors, which are commonly linked to host adaptation and probiotic functionality rather than pathogenicity. Importantly, no typical high-risk pathogenic determinants—such as Shiga toxin, cholera toxin, or core effector proteins of type III/type VI secretion systems—were detected. The absence of these well-characterized virulence factors further supports the non-pathogenic nature of this strain. RGI analysis against the CARD database identified strict hits (*vanT* and *vanY*) on the chromosome, with matching region identities up to 36.76%. These low identity values suggest that the detected sequences do not represent complete acquired vancomycin resistance genes; moreover, no intact vancomycin resistance operon (such as the *vanA* or *vanB* gene cluster) was detected in the genome sequence. This finding is consistent with previous reports [37] regarding vancomycin resistance-related genes in the genus *Leuconostoc*. Notably, no resistance genes were detected on the two plasmids, further supporting the low risk of horizontal transfer of antimicrobial resistance. Collectively, the genomic safety assessment supports the biosafety of *L. falkenbergense* SBL-85-2. The strain lacks typical high-risk virulence factors and horizontally transferable resistance genes, indicating its potential as a candidate for probiotic or functional food development.

Previously, *Streptomyces pluripotens* [38] and *Bacillus subtilis* [39] served as common antimicrobials against *A. hydrophila*, whereas *L. falkenbergense* SBL-85-2 was confined to food fermentation and preservation [22]. Here, we demonstrate that a BLIS produced by *L. falkenbergense* SBL-85-2 exhibits unique and highly effective antibacterial activity against *A. hydrophila* ATCC 7966. Furthermore, this BLIS exhibits broad-spectrum antimicrobial activity against both Gram-negative and Gram-positive bacteria, as well as significant antimicrobial activity against several important aquaculture-derived pathogens, such as *A. rivipollensis*, *A. salmonicida*, and *V. fluvialis*. This demonstrates the potential of *L. falkenbergense* SBL-85-2 as an antimicrobial agent against the aquaculture-derived pathogen *A. hydrophila* ATCC 7966.

The growth kinetics of *L. falkenbergense* SBL-85-2 show that as the bacteria grow, the pH of the culture medium continuously decreases and eventually stabilizes at 3.0. Its antibacterial activity begins during the logarithmic growth phase and peaks at 16 h, indicating that the accumulation of antibacterial substances is closely related to bacterial proliferation and the formation of a low-pH environment. The antimicrobial substances produced by this strain are stable and capable of exerting a sustained, potent inhibitory effect; their mechanism of action may be attributed to the synergistic effects of organic acids and BLIS [40]. Antimicrobial experiments against *A. hydrophila* ATCC 7966 strongly demonstrate that the metabolites secreted by SBL-85-2 exert potent inhibitory effects on the target pathogen.

A comprehensive evaluation shows that BLIS S-2 exhibits excellent stability under a variety of complex conditions and has high potential for antimicrobial applications [41]. The antimicrobial activity of BLIS S-2 decreases significantly as pH increases, a phenotype resembling that of typical Class II bacteriocins reported in the literature [42]. Class II bacteriocins typically possess a net positive charge due to their high content of basic amino acid residues, which facilitates their initial electrostatic interaction with negatively charged bacterial membranes [43]. We hypothesize that the reduced activity observed under alkaline conditions may be attributed to either a decrease in the positive charge density of the peptide or conformational changes that affect its membrane-interacting capacity. However, the detailed molecular mechanisms underlying this pH-dependent activity loss remain to be elucidated. In contrast, BLIS S-2 retains good antibacterial activity after heat treatment, demonstrating typical thermal stability, which is attributed to the small molecular weight, simple structure, and absence of complex post-translational modifications characteristic of Class II BLIS [44]. The thermal tolerance of BLIS S-2 may stem from the thermodynamically stable core provided by its N-terminal YGNGV motif. Even if the C-terminal amphiphilic α-helix undergoes transient, reversible unfolding at high temperatures, the rigid N-terminal framework ensures that it folds correctly during the refolding process, thereby maintaining its membrane-targeting function [45]. Protease sensitivity assays further confirmed the protein nature of BLIS S-2 [46]. BLIS S-2 exhibited significant sensitivity to papain and proteinase K, which have broad substrate spectra, as well as to trypsin and pepsin, which target key residues. However, it exhibits relative resistance to Pronase E. This difference is presumed to be related to the unique spatial folding of the N-terminal YGNGV motif of this bacterium [47]; this conformation effectively shields the cleavage site from Pronase E, whereas trypsin and pepsin specifically target its key active residues, leading to inactivation. Furthermore, BLIS S-2 exhibits good tolerance to continuous UV irradiation. This is primarily attributed to its stable spatial conformation, the absence of photosensitive key active sites, and a possible ability to resist oxidative damage, enabling it to withstand UV-induced oxidative damage [48].

The Pediocin gene cluster was identified in the SBL-85-2 strain in this study. Genetic dissection of this cluster suggests that its organization is characterized by a bacteriocin precursor as the core, integrated with various modules that may be associated with environmental adaptation. orf00028 is annotated as the Pediocin precursor peptide, which serves as the potential structural basis for the antibacterial activity of this strain. Notably, no genes encoding immunity proteins (such as PedB/PedC) or an ABC transporter system (PedD), which are typically tightly linked in a complete class II bacteriocin operon, were detected within this cluster. This absence suggests that orf00028 may not be able to independently form an active secretion system; its function might require trans-acting factors from other genomic loci, or alternatively, the gene may have become pseudogenized in the current host. Surrounding this core, the cluster contains a number of accessory genes potentially involved in stress responses and nutrient acquisition. Specifically, orf00020 (copper-efflux P-type ATPase), orf00034 (cadmium-transporting ATPase), orf00021 (metal-regulated DNA-binding protein MrgA), and orf00024 (inner membrane metal ion efflux transporter YrkD) together constitute a putative heavy metal resistance network. This network may confer tolerance to divalent cations such as Cu^2+^ and Cd^2+^, potentially helping the strain to withstand metal ion stress in industrial fermentation or intestinal environments. Meanwhile, orf00031, orf00032 (glycerol channel protein GlpF), and orf00038 (glycerol-3-phosphate transporter GlpT) may act synergistically to enhance glycerol uptake and utilization, potentially providing a competitive advantage in glycerol-rich niches. Additionally, orf00005 (NADH oxidase), orf00027 (thioredoxin), and orf00041 (acyl-CoA dehydrogenase) may participate in maintaining intracellular redox balance and fatty acid β-oxidation, which could help alleviate metabolic stress. At the regulatory level, orf00003, orf00014, and orf00018 jointly encode a putative Flp-type transcriptional regulator, which may suggest that gene expression within this region could be subject to complex regulatory cascades in response to environmental signals. In terms of genetic mobility, the dense distribution of transposase genes belonging to three distinct families—IS905 (orf00002), IS904 (orf00010), and IS3 (orf00015)—within this cluster suggests that this region may represent a genomic island or a mobile plasmid region acquired through horizontal gene transfer. The chimeric assembly of these functional modules may indicate that the host has integrated fitness-related genes from multiple sources through transposition events over long-term evolution, which may synergistically enhance its colonization and competitive abilities in specific microecological niches.

Results from dialysis and Tricine-SDS-PAGE analysis indicate that the purified BLIS S-2 produced by *L. falkenbergense* SBL-85-2 is a low-molecular-weight BLIS (<2.7 kDa). Its molecular weight is consistent with that of most reported BLIS derived from LAB (such as GA15 (4.3 kDa) [49] and G(2) (2.2 kDa) [50]). Given that BLIS production by *L. falkenbergense* remains largely uncharacterized, and Leucocin A (3.9 kDa) from the same genus has been extensively studied [26], the discovery of BLIS S-2 enriches the molecular-weight spectrum of BLIS from this species. It is worth noting that BLIS S-2′s smaller molecular weight confers a unique advantage against *A. hydrophila*: on the one hand, its low molecular weight enables BLIS S-2 to penetrate the extracellular polysaccharide barrier and biofilm matrix secreted by *A. hydrophila*, allowing it to more effectively contact and target the pathogen’s cell membrane [51]; On the other hand, owing to its low molecular weight, BLIS S-2 exhibits superior diffusivity and physicochemical stability compared to large-molecule proteins, suggesting its potential applicability in the complex aquatic environment and as a feed additive for aquatic animals [52]. Furthermore, it is readily degraded by endogenous proteases, making it unlikely to accumu0late as residues, thereby meeting the urgent need of the aquaculture industry for the development of highly effective, low-toxicity, novel antimicrobial alternatives.

Based on a combination of fluorescence staining, ultrastructural observations, and biochemical analysis, this study demonstrated from multiple perspectives that BLIS S-2 mediates the death of *A. hydrophila* ATCC 7966 by disrupting cell membrane integrity. After 2 h of treatment with BLIS S-2, the red fluorescence of the bacterial cells significantly increased, indicating that their cell membrane integrity had been compromised. This finding is consistent with the detection of the observed reduction in intracellular ATP levels, which decreased by 69.16 ± 4.10% at 2 h post-treatment, suggesting that membrane damage leads to the leakage of cellular contents. SEM results further supported this mechanism at the morphological level: the surfaces of BLIS S-2-treated cells exhibited roughening, pore formation, and leakage of cellular contents, providing direct visual evidence of membrane disruption. Taken together, these results indicate that the antibacterial activity of BLIS S-2 is associated with its capacity to damage the cell membrane, ultimately leading to cell lysis and death [53]. The current data support the conclusion that disruption of cell membrane integrity is a key mechanism by which BLIS S-2 inhibits the growth of *A. hydrophila*.

Several limitations of the present study should be acknowledged. First, BLIS S-2 was characterized as a crude preparation rather than a purified single compound. Although Tricine-SDS-PAGE and whole-genome sequencing provided suggestive evidence, the active peptide has not yet been subjected to structural confirmation by methods such as MALDI-TOF-MS, LC-MS/MS, or peptide sequencing. Therefore, we propose that BLIS S-2 is a Class II bacteriocin. Second, MIC and MBC values were not determined in this study, as we were unable to obtain a purified form of the peptide. Accordingly, the antibacterial efficacy in this study was primarily assessed qualitatively and semi-quantitatively through inhibition zone diameters and growth inhibition kinetics. Third, all experiments were conducted under in vitro conditions, and whether the results can be extrapolated to the in vivo environment remains unclear. Various complex factors in fish, including the immune system, gut microbiota, and metabolic clearance, may influence the actual antibacterial efficacy of BLIS S-2 in vivo; thus, its potential for in vivo application warrants further investigation. Future studies may explore appropriate delivery strategies to enhance the stability and bioavailability of this peptide for practical applications. Combination with other antimicrobial strategies could also be considered in future investigations to potentially achieve synergistic effects [54]. Nevertheless, this study reports the antibacterial activity of a BLIS derived from *L. falkenbergense* against *A. hydrophila* and provides preliminary evidence for its membrane-damaging mechanism. Future research will focus on purification and structural identification of the active compound, and further evaluate its application potential through in vivo infection models.

## 5. Conclusions

In summary, this study systematically characterized a low-molecular-weight BLIS S-2 derived from *Leuconostoc falkenbergense* SBL-85-2. Hemolytic activity assays showed no hemolytic zone on blood agar plates, and whole-genome prediction further revealed no typical high-risk virulence factors, with only low-identity vancomycin-related intrinsic genes (*vanT* and *vanY*) identified by the CARD database, suggesting that the producing strain may possess favorable in vitro biosafety. Additionally, whole-genome sequencing analysis further predicted that it produces a class II bacteriocin. BLIS S-2 exhibited broad-spectrum antimicrobial activity against various Gram-negative and Gram-positive bacteria, with particularly significant inhibitory effects against the aquatic pathogen *Aeromonas hydrophila* ATCC 7966. Stability tests revealed that the substance retained full antimicrobial activity after heat treatment at 100 °C for 1 h and continuous UV irradiation for 3 h; however, its activity decreased markedly with increasing pH, and it was sensitive to trypsin, papain, pepsin, and proteinase K, confirming its proteinaceous nature. Dialysis and Tricine-SDS-PAGE analysis identified BLIS S-2 as a low-molecular-weight antimicrobial peptide with an estimated molecular weight of less than 2.7 kDa. Mechanistic investigations using live/dead cell staining, scanning electron microscopy, and intracellular ATP measurements suggested that BLIS S-2 may exert its bactericidal effect by disrupting bacterial cell membrane integrity. Overall, the BLIS S-2 characterized in this study through a series of in vitro experiments provides preliminary experimental evidence for reducing antibiotic dependence and developing safe and effective alternative strategies; however, its practical application potential as an antibiotic alternative in aquaculture still requires further in vivo validation.

## Figures and Tables

**Table 1:** Antimicrobial spectrum of BLIS S-2 produced by *L. falkenbergense* SBL-85-2.

Indicator Strain	Source	Gram	Inhibition Zone (mm)
*A. hydrophila*	ATCC 7966	G^−^	39.14 ± 0.65
*A. rivipollensis*	MZ-17	G^−^	37.76 ± 0.47
*A. salmonicida*	XN-9	G^−^	36.48 ± 0.40
*E. coli*	ATCC 25922	G^−^	33.85 ± 0.38
*S. Typhimurium*	ATCC 14028	G^−^	27.92 ± 0.31
*S. aureus*	ATCC 25923	G^+^	32.68 ± 0.60
*V. fluvialis*	ZXL-80	G^+^	34.05 ± 0.32

Note: The diameter of the antibacterial zone covers the diameter of the Oxford cup. Data are presented as mean ± SD (*n* = 3).

## Data Availability

The original contributions presented in this study are included in the article and Supplementary Materials. Further inquiries can be directed to the corresponding authors.

## Supplementary Materials

The following supporting information can be downloaded at https://www.mdpi.com/article/10.3390/vetsci13090933/s1, Figure S1: Original full-length gel image for Figure 5, lanes M, 1, and 2.

## Abbreviations

The following abbreviations are used in this manuscript:

BLIS	Bacteriogen-like Antibacterial Substance
*A. hydrophila*	*Aeromonas hydrophila*
*L. falkenbergense*	*Leuconostoc falkenbergense*
*A. rivipollensis*	*Aeromonas rivipollensis*
*A. salmonicida*	*Aeromonas salmonicida*
*E. coli*	*Escherichia coli*
*S. Typhimurium*	*Salmonella Typhimurium*
*S. aureus*	*Staphylococcus aureus*
*V. fluvialis*	*Vagococcus fluvialis*
MAS	Motile Aeromonas Sepsis
AMR	Antimicrobial Resistance
LAB	Lactic Acid Bacteria
MDR	Multidrug-Resistant
GRAS	Generally Recognized As Safe
SEM	Scanning Electron Microscopy
PCR	Polymerase Chain Reaction
PBS	Phosphate-Buffered Saline
CFS	Cell-Free Supernatant
MWCO	Molecular Weight Cut-Off
BHI	Brain Heart Infusion
MH	Mueller-Hinton
LB	Luria–Bertani
PI	Propidium Iodide
ANOVA	Analysis Of Variance
